# Research Progress, Safety Regulation and Application Prospects in Health Food Development of Red Yeast Rice-Derived Bioactive Compounds: A Critical Narrative Review

**DOI:** 10.3390/foods15071146

**Published:** 2026-03-27

**Authors:** Xuan Chen, Meie Zheng, Qin Chen, Shun Wang, Xiwu Jia, Wangyang Shen, Mengzhou Zhou, Dongsheng Li

**Affiliations:** 1 School of Life and Health Sciences, Hubei University of Technology, Wuhan 430068, China; chenxuan_1986.163@163.com; 2 Hubei Key Laboratory of Resource Utilization and Quality Control of Characteristic Crops, College of Life Science and Technology, Hubei Engineering University, Xiaogan 432000, China; meiezheng@hbeu.edu.cn; 3 School of Food Science and Engineering, Wuhan Polytechnic University, Wuhan 430023, China; immmortal1201@163.com (Q.C.); wangshun7914@163.com (S.W.); jiaxiwu212@gmail.com (X.J.); swy.3@163.com (W.S.)

**Keywords:** red yeast rice (RYR), *Monascus purpureus*, monacolin K (MK), functional food, citrinin, regulatory frameworks

## Abstract

Red yeast rice (RYR), a traditional fermented product obtained via rice fermentation with *Monascus purpureus*, has a millennia-long history of culinary and medicinal use in East Asia and has gained global attention as a prominent functional food ingredient for its well-recognized cholesterol-lowering properties. This review is driven by one core question: How can the dual challenges of standardizing key bioactive constituents, particularly monacolin K (MK), while eliminating the mycotoxin citrinin be addressed through biotechnological and analytical advances? This narrative review consolidates the latest research progress on RYR-derived bioactive compounds, with a specific focus on their production optimization, multifaceted health-promoting potentials, safety regulation, and application prospects in health food development. We elaborate on key advances in fermentation biotechnology and strain engineering for enhancing the yield of the core lipid-lowering component MK while eliminating the nephrotoxic mycotoxin citrinin, and comprehensively summarize the synergistic bioactivities of RYR metabolites beyond MK. The current applications of RYR in functional foods, dietary supplements, and traditional fermented products are detailed, alongside a comparison of the divergent regulatory frameworks for RYR across major global markets. Finally, we identify critical bottlenecks restricting RYR industrialization, including extreme inter-product heterogeneity and global regulatory fragmentation, and propose evidence-based future research directions to facilitate the development of safe, standardized, and effective RYR-based health foods.

## 1. Introduction

Red yeast rice (RYR), known as hongqu in China, is a unique fermentation product with a rich history deeply rooted in the culinary and medicinal heritage of East Asia [1]. It is produced by the solid-state fermentation (SSF) of cooked rice using specific filamentous fungi, most notably *Monascus purpureus*—a process that dates back at least two millennia in Chin [2]. Throughout its long history, RYR has fulfilled dual roles: as a food ingredient, its characteristic red pigments serve as natural colorants and flavor enhancers, lending a distinctive hue and subtle taste to a wide range of traditional foods such as preserved meats, fish, fermented sauces, and rice wines [3].

Beyond its culinary uses (e.g., tofuyo, cured meat, steamed pork with rice flour), RYR occupies an esteemed position in Traditional Chinese Medicine [2]. Classical pharmacopeias, including the Ming Dynasty’s Compendium of Materia Medica, recorded its therapeutic benefits, describing its use to strengthen bodily functions, promote blood circulation, relieve digestive ailments, and fortify the spleen [1]. This longstanding ethnomedical application established RYR as a health-promoting agent well before the emergence of modern pharmacology and the concept of “functional foods”. Such historical recognition provides essential context for its contemporary revival and the growing scientific interest it continues to attract [1,3].

A pivotal scientific breakthrough in the late 20th century accelerated the transformation of RYR from an ancient remedy to a globally recognized nutraceutical [4]. Researchers identified that the primary lipid-lowering effects of RYR, its most widely acknowledged health benefit, are mainly due to a class of secondary metabolites referred to as monacolins [2]. Among these compounds, monacolin K (MK) is particularly noteworthy for being chemically and structurally identical to lovastatin, the initial statin drug sanctioned by the U.S. Food and Drug Administration for managing hypercholesterolemia [4]. This revelation established a solid pharmacological foundation for the traditional use of RYR in “promoting blood circulation” and propelled it into the global limelight [5].

The implications of this finding are substantial for global public health, given that elevated low-density lipoprotein cholesterol (LDL-C) is a well-established major modifiable risk factor for atherosclerotic cardiovascular disease, the leading cause of mortality worldwide [6]. The lipid-lowering efficacy of RYR has been robustly validated by a large body of high-quality clinical evidence: Trogkanis et al. [7] conducted a systematic review and meta-analysis of 32 randomized controlled trials and confirmed that RYR supplementation significantly reduced serum total cholesterol (TC), LDL-C and triglyceride levels in hypercholesterolemic populations, with a pooled effect size comparable to low-intensity statin therapy; Gerards et al. [8] further demonstrated in a meta-analysis that RYR extract enriched with MK could reduce LDL-C by an average of 1.02 mmol/L in subjects with mild-to-moderate hypercholesterolemia. These findings have firmly established RYR as one of the most effective and widely applied functional ingredients and dietary supplements for the management of dyslipidemia [7]. Notably, RYR has gained extensive popularity among individuals intolerant to prescription statins or those preferring natural alternatives for cholesterol management [6], as Cicero et al. [9] verified in a multi-center clinical trial that RYR is a safe and effective lipid-lowering option for statin-intolerant patients, with no severe adverse events reported during 12 weeks of intervention; Liasi et al. [10] also confirmed that even low-dose MK (3 mg/day) from RYR could exert significant cholesterol-lowering effects in this population.

However, the scientific narrative and health benefits of RYR extend far beyond MK alone. Intensive metabolomic investigations have demonstrated that RYR fermentation produces a complex and diverse metabolome with a wide array of bioactive constituents beyond monacolins [1]. Zhu et al. [1] characterized the full metabolomic profile of RYR via UHPLC-QToF-MS and identified more than 50 bioactive compounds, while Liu et al. [2] further summarized the full spectrum of secondary metabolites produced by *Monascus purpureus* during fermentation. In addition to monacolins, RYR contains abundant functional compounds including characteristic *Monascus* pigments (Mps),GABA, sterols, isoflavones and polysaccharides, each of which contributes to a broad spectrum of pharmacological activities as verified by multiple independent research teams [11]. Specifically, Chen et al. [12] and Alqurashy et al. [13] confirmed that Mps exert potent antioxidant, anti-inflammatory and hepatoprotective effects; Wu et al. [14] demonstrated that *Monascus*-derived GABA has significant antihypertensive and neuroprotective activities; and Li et al. [15] verified that RYR polysaccharides have immunomodulatory and prebiotic properties. Emerging evidence, predominantly from in vitro and in vivo animal studies, indicates that these components exert synergistic interactions to drive the overall health benefits of RYR [1], with Zhou et al. [16] further revealing that the combination of MK and *Monascus* pigments could synergistically ameliorate lipid metabolic disorders and gut microbiota dysbiosis in high-fat diet-fed rats, which is a core mechanism underlying the holistic health effects of RYR beyond single MK supplementation. This growing body of multi-dimensional evidence strongly supports the application of RYR as a multifunctional functional ingredient for daily health maintenance and chronic disease prevention [7].

The clinical efficacy of RYR is derived from the presence of pharmacologically active, drug-like compounds within a natural food matrix, which creates a fundamental paradox that places RYR at the intersection of scientific research, commercial development and global regulatory governance [4]. This inherent dual identity as both a traditional food and a pharmacologically active substance has led to a complex and often inconsistent landscape in both scientific research and regulatory oversight worldwide [17]. Despite its global popularity, the development and safe application of RYR still face three core critical challenges, all of which have been widely documented by multiple research teams and regulatory authorities. First, extreme inter-product heterogeneity in the chemical composition and active ingredient content of commercial RYR products: Avula et al. [18] and Righetti et al. [19] independently reported that the MK content in commercial RYR raw materials and supplements ranges from undetectable to 78.4 mg/g dry weight, with a 40-fold variation in MK content across different supplement products, leading to unpredictable therapeutic effects and safety risks. Second, the potential contamination of citrinin, a nephrotoxic mycotoxin produced during *Monascus* fermentation: the European Food Safety Authority (EFSA) [6] reaffirmed in its 2025 scientific opinion that citrinin contamination remains a major safety hazard for RYR products, with unoptimized fermentation processes leading to citrinin levels exceeding the EU maximum limit by up to 40-fold; Kamle et al. [20] also systematically summarized the toxicological risks of citrinin in RYR products. Third, the fragmented and inconsistent global regulatory framework for RYR, which creates significant uncertainty for consumers, clinicians and manufacturers [21], as detailed by Childress et al. [17] in their analysis of the divergent regulatory approaches between the US FDA, EU and China.

**Figure 1:**
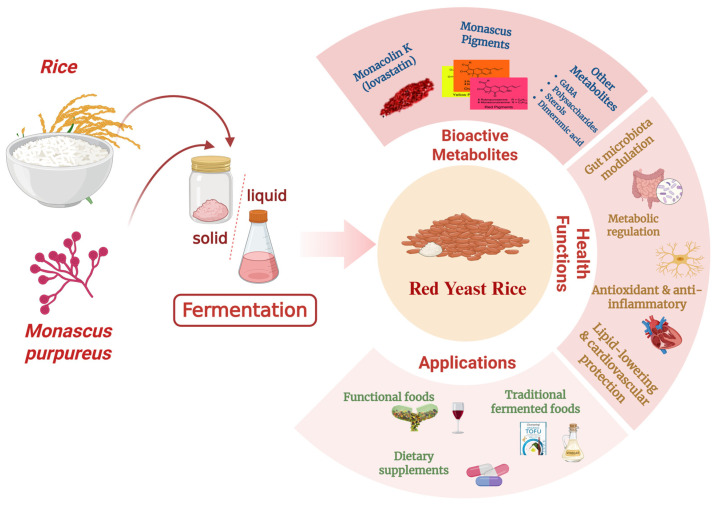
Key bioactive metabolites of RYR and their applications and health functions.

Therefore, this review is guided by one specific research question: how can the fundamental product-quality challenges—namely, the variability of MK content and the risk of citrinin contamination—be resolved through integrated advances in strain engineering, fermentation science, and detection technologies? Addressing this question is of critical pertinence for the future development of the field. The global burden of cardiovascular disease continues to rise, driving demand for accessible, preventive nutritional strategies (Figure 1). Simultaneously, the convergence of biotechnology, data science, and personalized nutrition is reshaping the functional food landscape, demanding higher standards of efficacy, safety, and substantiation. A precise analysis focused on reconciling its efficacy–safety paradox and regulatory identity is therefore essential. It will not only consolidate current knowledge but also provide a clear translational roadmap for developing reliable, next-generation RYR products that meet the rigorous demands of modern public health and personalized wellness.

## 2. *Monascus purpureus*: The Microbial Architect of RYR

### 2.1. Biological Origins, Taxonomy, and Characteristics

The distinctive properties of RYR stem from the metabolic actions of a specific group of filamentous fungi, notably *Monascus purpureus*, a mold belonging to the phylum Ascomycota, class Eurotiomycetes, and family Monascaceae [1]. While *Monascus purpureus* is the most commonly cited species, the *Monascus* genus is diverse, with other related species like *M. ruber* and *M. anka* also utilized in both traditional and industrial fermentations [1]. The choice of strains significantly influences the attributes of the end product, as distinct species, and even different strains within the same species, can yield varied lineages of pigments, citrinin, and other secondary metabolites.

Morphologically, *Monascus* species develop dense mycelial networks and primarily reproduce asexually through the formation of botryoid conidial clusters [1]. In laboratory settings, these fungi exhibit rapid growth on standard mycological media such as Potato Dextrose Agar, forming colonies typically displaying a distinctive reddish or purplish hue due to pigment release. Optimal growth and metabolite production generally occur within specific environmental conditions, with temperatures between 25 °C and 30 °C and relative humidity levels ranging from 65% to 85% being most conducive [1]. A thorough comprehension of these biological characteristics is crucial for effectively cultivating the fungus and enhancing fermentation processes to attain a product with the desired composition and bioactivity.

### 2.2. Fermentation Biotechnology

The cultivation method of *Monascus purpureus* serves as a critical control point that significantly impacts the biochemical profile, efficacy, and safety of the final RYR product. The selection of fermentation technology is not solely a matter of processing efficiency; it fundamentally influences the metabolic activity of the microorganism. This, in turn, shapes the concentration and ratio of beneficial bioactive compounds while also determining the potential presence of undesirable contaminants. Two primary fermentation systems are utilized in the production of RYR [3].

Solid-state fermentation (SSF) is the traditional and long-established production method. In this process, *Monascus* fungi are inoculated directly onto a solid, moist substrate, predominantly steamed rice [22,23]. The fungus grows on and penetrates the rice kernels, progressively consuming the starch and generating the diverse array of secondary metabolites that characterize the final product. SSF is often favored for its ability to replicate the natural growth environment of filamentous fungi, which can enhance yields of certain secondary metabolites, including specific pigments and enzymes. However, SSF presents significant technical challenges. The heterogeneous nature of the solid substrate hinders precise regulation of crucial parameters such as temperature, pH, moisture, and aeration throughout the fermentation process. This lack of control can lead to variability in product quality across batches and heightens the risk of contamination by undesirable microorganisms, potentially including citrinin-producing strains.

Submerged fermentation (SmF) serves as a contemporary, industrial-scale alternative to SSF. In this approach, fungi are cultivated in a sterile liquid nutrient medium within large, tightly controlled bioreactors [24,25]. SmF presents several advantages over SSF, including the ability to precisely monitor and regulate environmental conditions, thereby ensuring homogeneity and consistency in the process. These factors contribute to more predictable product yields, facilitate easier scale-up for mass production, and significantly reduce the risk of microbial contamination. Nevertheless, SmF has its limitations. The high-shear conditions produced by mechanical agitation in bioreactors can impose physical stress on fungal mycelia, potentially altering their metabolic behavior. 

Consequently, the metabolite profile obtained through SmF often differs markedly from that of SSF-derived products, which may lead to lower yields of specific target compounds or an altered ratio of bioactive constituents. This fundamental methodological distinction is a key factor contributing to the lack of standardization in the RYR market (Table 1). In contrast, SmF predominantly produces the lactone form MK, which has lower oral bioavailability and relies on hepatic carboxylesterase activation to exert lipid-lowering effects, resulting in attenuated and more variable therapeutic efficacy. SSF offers superior controllability to decouple MK biosynthesis from citrinin production. Multiple studies confirmed that optimized SSF systems (e.g., indica rice substrate, variable temperature regimes, pH modulation) achieved undetectable citrinin levels while maintaining high MK yields [26,27]. This is because SSF enables spatial and temporal regulation of fungal gene expression: low-pH conditions (≤5.0) in SSF significantly downregulate citrinin biosynthetic genes (*citS*, *citE*) without impairing MK synthesis. In the homogeneous liquid environment of SmF, the polyketide synthase pathways shared by MK and citrinin are coordinately regulated, making it extremely difficult to suppress citrinin production while enhancing MK titers, resulting in a far higher risk of citrinin exceeding global regulatory limits. SmF typically requires the addition of synthetic antifoaming agents, chemically defined carbon/nitrogen sources, and extraction solvents, introducing potential risks of chemical residue and impurity contamination. SSF, by contrast, uses natural edible grain substrates with no requirement for synthetic additives during fermentation, minimizing exogenous toxicant exposure. Furthermore, the aerial hyphal growth morphology in SSF reduces metabolic stress and the production of unwanted toxic secondary metabolites, compared to the mycelial pellet growth form in SmF, which is more prone to non-target toxic metabolite accumulation under shear stress and homogeneous culture conditions. As a result, two products labeled as “RYR” may exhibit significantly different therapeutic and safety profiles.

*Monascus purpureus*-derived fermented products are valued for bioactive metabolites, particularly MK—a potent cholesterol-lowering agent targeting HMG-CoA reductase—and constrained by the nephrotoxic mycotoxin citrinin. The biosynthesis of these metabolites is predominantly regulated by fermentation modalities and physicochemical parameters, making strategy optimization critical for safe and high-yield MK production.

SSF consistently outperforms SmF in MK biosynthesis, with a maximum MK titer of 40.2 mg/g achieved using coix seed and glutenin as substrates—far exceeding yields from conventional SMF systems [28]. This superiority stems from enhanced oxygen mass transfer, a physiologically relevant microenvironment for fungal growth, and sustained precursor supply inherent to SSF [27].

**Table 1:** Comparison of fermentation characteristics, MK and citrinin production among different *Monascus* fermentation systems.

Substrate	Fermentation Mode	Strain	Key Fermentation Conditions	Monacolin K (MK) Yield	Citrinin Yield	Major Co-Products	Reference
Indica rice, japonica rice, millet, red rice, brown rice, black rice, glutinous rice, early indica rice	Solid-state fermentation (SSF)	*Monascus purpureus* MS-12 (mutagenized by ARTP + heavy ion beam irradiation; parental strain *Monascus purpureus* LQ-6)	30 °C, dark fermentation for 7 d; inoculum size 10% (*v*/*v*), initial moisture content ~55% (*w*/*w*); static cultivation for the first 2 d, mixing every 12 h thereafter, 0.2% acetic acid solution supplemented to maintain moisture from day 3	Not reported	Not detected (ND)	*Monascus* pigments (MPs), maximum 4526 U/g with indica rice as substrate	[15]
Rice, Pueraria, yam (dry mass ratio 8:1:1)	Mixed solid-state fermentation (SSF)	*Monascus ruber*	30 °C, fermentation for 15 d; inoculum size 10^6^ spores/mL	1.40 ± 0.04 mg/g	Not reported	Total flavonoids (max. 16.36 mg/g); daidzein (97% increase); diosgenin (175% increase)	[29]
Rice-based solid-state fermentation medium	Solid-state fermentation (SSF)	1. *M. pilosus* MS-1 (wild type, WT); 2. A1 (Δ*pigA*, WT background); 3. C1 (acc overexpression, WT background); 4. C2 (acc overexpression, A1 background); 5. H1 (*hos2* overexpression, WT background)	28 °C, fermentation for 14 d	WT: 15.8 mg/g;A1: 20.3 mg/g (+28.5% vs. WT); C1: 18.1 mg/g (+14.7% vs. WT); C2: 24.5 mg/g (+43.9% vs. WT); H1: 25.2 mg/g (+36.1% vs. WT)	Not reported (parental strain MS-1 is citrinin-free)	*Monascus* azaphilone pigments (MonAzPs)	[15]
Northeast pearl rice (supplemented with 1.2 g yeast extract, 1.6 g peptone, 1.2 g glycerol per 25 g dry rice)	Co-culture solid-state fermentation (SSF)	1. *Monascus purpureus* R5 (monoculture); 2. Lovastatin-producing microbial co-culture system (LPMCS): *Monascus purpureus* R5 + *Lacticaseibacillus casei* S5 + *Saccharomyces cerevisiae* J7	30 °C, optimized fermentation time 13.88 d; initial moisture content 50.5% (*w*/*w*), inoculum ratio 10.27% (v/w), initial pH 5.0, cell age 5 d, loading quantity 25 g/250 mL	Monoculture R5: 4.82 mg/g; Co-culture LPMCS: 7.43 mg/g (+54.21% vs. monoculture)	0.065 μg/mL (far below the Chinese national standard limit of 80 μg/mL)	Not reported	[30]
Indica rice (supplemented with glucose 50 g/L, peptone 20 g/L, MgSO_4_ 0.5 g/L, KH_2_PO_4_ 1 g/L)	Variable-temperature solid-state fermentation (SSF)	*Monascus purpureus* HNU01	Initial pH 5.5, initial moisture content 40% (*w*/*w*); variable temperature: 30 °C for the first 3 d, then 24 °C for 15 d (total 18 d); 10% (*w*/*w*) sterile water supplemented on day 4	9.5 mg/g (acid-form MK accounted for up to 90% of total MK)	Not detected (ND)	Not reported	[27]
Coix seed + gluten fractions (glutenin, urea-soluble glutenin, αβγ-gliadin, ω-gliadin, alkali-soluble glutenin); optimal substrate: coix seed + glutenin (dry mass ratio 1:1, with deionized water at 1:1:2 g/g/v)	Solid-state fermentation (SSF)	*Monascus purpureus* M3 (screened from commercial red yeast rice; other tested strains: M1, M2, M4, M5)	32 °C, fermentation for 27 d; substrate sterilized at 110 °C for 40 min	40.2 mg/g (5.71-fold higher than original commercial red yeast rice)	Not reported	Not reported	[28]

Substrate composition is a pivotal determinant of metabolite profiles. Indica rice emerged as the optimal substrate for SSF, supporting 4526 U/g of *Monascus* pigments and undetectable citrinin in a mutagenized *Monascus purpureus* strain [26]. In contrast, colored (red, brown, black) and glutinous rice downregulated MK and pigment biosynthetic gene clusters, attenuating product yields. This discrepancy is attributed to indica rice’s favorable amylose–amylopectin ratio (13–15% amylose/total starch) and balanced carbon-to-nitrogen ratio (~10:1), which promote metabolic flux toward MK synthesis [26]. Mixed substrates (rice:Pueraria:yam = 8:1:1) further elevated MK content to 1.40 mg/g via synergistic nutrient provision [29].

Physicochemical parameters directly modulate secondary metabolism. A variable temperature regime (30 °C for 3 days, 24 °C for 15 days) boosted MK to 9.5 mg/g with no citrinin detected, aligning with fungal growth phases—high temperatures support initial mycelial proliferation, while lower temperatures favor secondary metabolite synthesis [27]. Optimized initial pH (5.5), moisture content (40%), and inoculum ratio (10.27%) in a *Monascus purpureus*-probiotics co-culture system increased MK yield by 54.21% [30], as probiotics enhance nutrient bioavailability. Notably, low pH (≤5.0) suppresses citrinin by downregulating *citS* and *citE* genes while preserving MK synthesis [26].

Strain modification further enhanced MK production and citrinin elimination. ARTP-heavy ion mutagenesis yielded *Monascus purpureus* CSUFT-1 with 1.67-fold higher MK and no citrinin [31]. Metabolic engineering (e.g., *pigA* knockout, *acc*/*hos2* overexpression) increased MK by up to 43.9% via redirected metabolic flux and histone acetylation modulation [15]. RT-qPCR revealed upregulation of key MK genes (e.g., *mokC*: 6.8-fold, *mokF*: 22-fold) in early fermentation, with sustained *mokE* overexpression maintaining pathway activity [31].

Collectively, integrating substrate selection, process optimization, and strain engineering enables efficient MK production with minimal citrinin. These strategies address industrial bottlenecks, providing scalable protocols for safe red yeast rice production and advancing *Monascus*-fermented products in functional foods and pharmaceuticals.

One-to-one quantitative matching analysis was conducted for each core metabolite of RYR, with objective delineation of both clinically and regulatory aligned production scenarios and critical mismatches between production yields and established therapeutic, safety, or regulatory dose thresholds across all target analytes. For MK, the primary lipid-lowering bioactive constituent of RYR, the 10 mg/day therapeutic effective dose endorsed by the European Food Safety Authority (EFSA) [32] is achievable with a daily intake of 0.5–5.0 g of commercial RYR raw material, a quantity readily incorporated into standard oral supplement formats; however, the extreme inter-product variability in commercial MK yields (0.45–78.4 mg/g) means that a fixed 1 g daily dose of RYR can deliver between 4.5 mg and 78.4 mg of MK, resulting in either failure to reach the therapeutic threshold or extreme exceedance of the 3 mg/day safety threshold reaffirmed by EFSA [33] for severe adverse musculoskeletal and hepatic events. In the case of GABA, a secondary functional metabolite frequently cited for antihypertensive activity, the established 10–20 mg/day clinically effective antihypertensive dose requires a daily RYR intake of 3–67 g given the average commercial GABA yield of 0.3–3.0 mg/g, a quantity impractical for standard supplement formulations, indicating that GABA from conventional RYR products rarely contributes to clinically relevant physiological effects. Finally, for the nephrotoxic mycotoxin citrinin, unoptimized RYR production with an average citrinin yield of 2000 μg/kg results in a 40-fold exceedance of the European Union (EU) [34] maximum permitted limit (MPL) of 100 μg/kg at a 2 g daily RYR intake, whereas optimized strain selection and fermentation process control maintain citrinin levels in full compliance with global regulatory requirements.

A well-characterized set of specific production conditions that drive citrinin biosynthesis and accumulation must be strictly avoided to ensure the safety and regulatory compliance of RYR products, with robust mechanistic and industrial evidence supporting each control threshold. At the pre-fermentation stage, the use of unmodified wild-type *Monascus* strains with intact citrinin biosynthetic gene clusters, substrate formulations with a carbon-to-nitrogen ratio < 10:1, and excess inorganic nitrogen sources must be avoided, as these factors significantly upregulate the core toxin synthesis genes *pksCT*, *citS*, and *citE*, increasing citrinin titers by up to 10-fold even in low-toxin strains. During fermentation, the most critical high-risk conditions requiring strict avoidance include sustained neutral-to-alkaline pH (≥5.5) in the mid-to-late production stage, constant high-temperature fermentation (≥32 °C for the full production cycle), hypoxic conditions caused by excessive substrate compaction or >60% initial moisture content in solid-state fermentation, and extended fermentation duration (>21 days). Each of these conditions preferentially shifts *Monascus* secondary metabolism toward citrinin production, while their avoidance can reduce citrinin to undetectable levels without impairing MK biosynthesis, as validated in multiple independent industrial and laboratory studies. For post-harvest processing and storage, high-temperature drying (>60 °C), alkaline extraction conditions, and long-term ambient aerobic storage must be avoided, as these practices either increase relative citrinin content via MK degradation or drive de novo toxin accumulation via residual fungal growth. The failure to avoid these high-risk conditions is the primary driver of citrinin non-compliance in commercial RYR products globally, as highlighted in the 2025 EFSA scientific opinion, yet no harmonized international standards exist to mandate these critical control measures for RYR production.

### 2.3. Strain Engineering in Monascus purpureus

Strain engineering in *Monascus purpureus* has emerged as a powerful tool to optimize secondary metabolite production and mitigate toxic byproducts, with CRISPR/Cas9-based technologies dominating recent advances. Key applications focus on enhancing valuable metabolites (MK, Mps) and reducing citrinin, a nephrotoxic mycotoxin (Table 2). CRISPR/Cas9-mediated gene knockout and overexpression have been widely used to target regulatory genes: disruption of *comp54181_c0* (a negative regulator) increased MK yield by 11.9% and pigments by up to 48% [35], while knockout of *rtt109* (histone acetyltransferase) boosted pigments (37.43–42.32%) but unexpectedly increased citrinin (34.54%) [36]. Targeting citrinin synthesis genes showed promising results: *ctnA* knockout reduced citrinin to 22% of wild type [37], *ctnD* editing decreased citrinin by over 98% [38], and *CtnC* overexpression lowered citrinin by 12.93–67.34% [39]. For pigment enhancement, CRISPR inactivation of negative regulators *MpigI* and *MpigI′* in *M. ruber* achieved 2.5–18.5-fold increases in Mps [40]. Advanced base editors like MIDBE facilitated random genomic evolution, improving MK yield by 977.1% [41]. These strategies demonstrate precise metabolic manipulation, but balancing beneficial metabolites and reducing toxins remains critical for industrial applications.

## 3. Bioactive Components in RYR and Their Health Potential

### 3.1. Monacolins

Monacolins constitute a distinct class of polyketide-derived secondary metabolites generated during the fermentation of RYR. Among these, MK (also known as lovastatin) is the most representative and has been studied most extensively. A defining feature of MK is its lactone moiety, which can exist in a closed (lactone) or open (β-hydroxy acid) conformation. Under neutral to alkaline conditions, the β-hydroxy acid form predominates and exerts potent inhibitory activity against hydroxy methylglutaryl coenzyme A(HMG-CoA) reductase, thereby effectively blocking cholesterol biosynthesis and modulating systemic lipid metabolism [42,43]. Beyond MK, additional congeners such as monacolins J, L, and X have been identified. These analogs exhibit subtle variations in side-chain architecture and functional groups, modifications that can markedly influence their pharmacokinetics, bioavailability, and lipid-lowering efficacy [44]. The structural diversity within this compound family not only reflects the biosynthetic versatility of *Monascus* species but also suggests that mixtures of monacolins, as found in RYR preparations, may exert synergistic or additive effects. MK exhibits a broad spectrum of pharmacological activities, the majority of which are preclinical, encompassing anticancer, neuroprotective, anti-inflammatory, hepatoprotective, antidiabetic, and lipid-lowering effects, as well as the attenuation of atherosclerotic progression [45,46,47]. These properties highlight its considerable potential as a multifunctional agent for disease prevention and therapeutic intervention.

**Figure 2:**
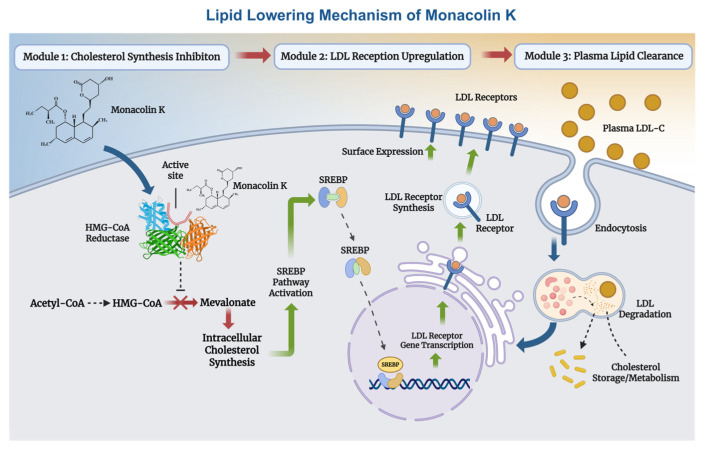
Lipid Lowering Mechanism of Monacolin K.

One of the principal physiological functions of MK is the reduction in cholesterol. Mechanistically, it modulates key enzymes and transcription factors involved in lipid metabolism, including regulators of high-density lipoprotein cholesterol (HDL-C) and LDL-C and apolipoprotein B. Through these actions, MK inhibits the synthesis of cholesterol, triglycerides, and fatty acid oxidases, while simultaneously reducing intestinal LDL-C absorption and promoting cholesterol transport (Figure 2).

Clinical evidence further supports these mechanistic insights. A systematic review by Liasi et al. [10] demonstrated that MK exerts significant cholesterol-lowering effects even at relatively low doses (3 mg/day), particularly when administered in combination with other dietary ingredients. Complementarily, Gerard et al. [8] reported that RYR extract enriched with MK reduced LDL-C levels by 1.02 mmol/L, highlighting its therapeutic potential as a safe and effective option for hypercholesterolemia management, especially in patients with statin intolerance, and underscoring its role in reducing cardiovascular risk.

Monacolin K (MK), the primary active hypocholesterolemic constituent of RYR, exerts significant lipid-modifying effects across various preparations, with substantial variability in standardized MK content and corresponding clinical outcomes [48,49,50]. For the standardized nutraceutical ROSSOPURO^®^ Forte, formulated with 4.5% total monacolins and 2.8 mg MK per capsule, a 3-month randomized, double-blind, placebo-controlled trial in subjects with mild dyslipidemia demonstrated a 19.3% reduction in LDL-C, an 8.3% reduction in TC, and a 29.3% elevation in HDL-C, with no significant alterations in TG or adverse events reported [48]. Xuezhikang, a purified RYR pharmaceutical preparation, contains 6 mg total monacolins (2.5 mg MK/lovastatin) per pill, with a daily 1200 mg dose delivering 10 mg MK [49]. In registrational trials, 8-week Xuezhikang intervention reduced LDL-C by 28.5%, TC by 23.0%, TG by 36.5%, and increased HDL-C by 19.6% in hyperlipidemic patients; the landmark China Coronary Secondary Prevention Study further confirmed 17.6% LDL-C, 10.9% TC, and 14.6% TG reductions over 4.5 years, alongside a 45% reduction in major coronary events [51]. Commercially available RYR supplements typically contain 2% MK, with daily doses of 3–10 mg MK per European regulatory limits. Meta-analyses of randomized controlled trials showed that such preparations reduced LDL-C by up to 20% (mean 1.02 mmol/L), TC by 1.0 mmol/L, and TG by 0.26 mmol/L, with efficacy comparable to low-intensity statins, while even low-dose RYR (2 mg/day MK) yielded significant LDL-C and TC reductions in mild dyslipidemia [48,49,50]. Collectively, the lipid-lowering efficacy of MK-containing products is tightly linked to standardized MK content, with most preparations exhibiting favorable safety profiles, supporting their clinical application in mild dyslipidemia and statin-intolerant populations [48,49,50].

MK has been extensively investigated for its lipid-lowering, anticancer, and neuroprotective activities, underscoring its broad therapeutic potential. Advancing mechanistic studies and well-designed clinical trials will be essential to fully elucidate its modes of action, optimize its efficacy, and ensure safety. Such efforts are expected to further enhance the translational value of MK, positioning it as a promising candidate at the interface of functional foods and pharmaceutical development.

### 3.2. Pigments

*Monascus* species synthesize a variety of valuable secondary metabolites, known as MPs. MPs are a structurally diverse group of azaphilone metabolites that are generally categorized into three major classes: yellow pigments (e.g., monascin (MS), ankaflavin (AK)), orange pigments (e.g., rubropunctatin (O1), monascorubrin (O2)), and red pigments (e.g., rubropunctamine (RP), monascorubramine (MB)) [52]. Although these pigments share a common pyranoquinone backbone, variations in side-chain substituents and subsequent reactions with amino acids or amines give rise to their distinct color groups. The biosynthesis of MPs is initiated by a type I polyketide synthase, which catalyzes the condensation of acetyl-CoA and malonyl-CoA to generate a hexanone chromophore. This intermediate undergoes esterification with medium- and long-chain fatty acids derived from fatty acid biosynthesis to yield orange pigments, which act as pivotal intermediates in pigment biosynthesis. Orange pigments are chemically versatile: they may react with nitrogen-containing compounds to form red pigments or undergo reduction to produce yellow pigments [53,54]. This dynamic interconversion illustrates the structural diversity and biochemical complexity of MPs biosynthesis. Among the three pigment groups, red pigments have attracted the greatest attention, as they are widely applied in food processing yet relatively rare among commercially available natural pigments. Red pigments produced by *Monascus purpureus* are considered promising natural pigments due to their wide chemical diversity and multiple biological activities, including immunomodulatory, antioxidant, and antitumor effects [55]. In contrast, yellow pigments are typically produced as relatively stable mixtures enriched in MS and AK, which have demonstrated potent antioxidant, anti-inflammatory, and lipid-lowering properties [56,57,58].

The stability of MPs is strongly influenced by environmental conditions. Acidic pH favors the stability of red pigments, whereas neutral to alkaline conditions shift the equilibrium toward yellow and orange forms. While MPs display moderate thermal stability—facilitating their application in various food products—they remain sensitive to light and oxidative degradation, which can limit shelf life [59,60]. Importantly, their characteristic structural features, particularly conjugated double bonds and quinone moieties, not only confer their vibrant coloration but also underlie many of their antioxidant and bioactive properties.

As early as 2010, Zheng et al. [61] reported that Rubropunctatin (RB), a predominant pigment derived from RYR, exerted pronounced antiproliferative effects against human gastric adenocarcinoma BGC-823 cells. RB inhibited cell proliferation with an IC_50_ of 12.57 μM, a potency comparable to that of paclitaxel at the same concentration. Moreover, Wei et al. [58] examined the effects of *Monascus purpureus* pigment extracts on cellular senescence and tumor cell growth. Both the crude extract, MK and fractions enriched in RB and MB demonstrated dose-dependent inhibitory activity against a hepatocarcinoma cell line. Notably, RB, the crude extract, and MK all suppressed cell growth, while treatment with RB and the crude extract significantly enhanced apoptotic cell accumulation. Collectively, these findings based solely on in vitro evidence suggest the promising in vitro anticancer activity of RYR pigments, especially RB, as multifunctional bioactive components.

Zhou et al. [16] investigated the regulatory mechanisms and lipid-lowering effects of different MPs (yellow, red, and orange) in rats fed a high-fat diet. Administration of these pigments at varying concentrations significantly reduced serum triglyceride levels, suppressed hepatic cholesterol accumulation, and alleviated lipid metabolism disorders. In addition, MPs modulated the composition of the intestinal microbiota, with certain beneficial taxa (such as *Oscillibacter* sp., *Ruminococcus albus*, *Clostridium* sp., etc.) showing negative correlations with serum and hepatic lipid markers. These findings, based mainly on animal studies, suggest that MPs not only improve lipid homeostasis but also exert prebiotic-like effects on gut microbial ecology, highlighting their potential, supported mainly by animal studies, as functional food ingredients for the prevention and management of hyperlipidemia and intestinal dysbiosis. Lee et al. [57] further demonstrated that the yellow pigments produced by *Monascus* fermentation, MS and AK, exert anti-obesity effects in high-fat diet-induced obese mice. These effects were primarily achieved by suppressing adipogenesis through downregulation of CCAAT/enhancer-binding protein β and its downstream targets, proliferator-activated receptor γ and CCAAT/enhancer-binding protein α, thereby inhibiting adipocyte differentiation. In addition, the pigments enhanced lipase activity while reducing heparin-releasable lipoprotein lipase activity, further contributing to the inhibition of lipid accumulation. Dong et al. [56] investigated the anti-glycation activities and underlying mechanisms of several MPs-AK, MS, O1, and O2-using a bovine serum albumin (BSA)-fructose model. All four pigments effectively inhibited the formation of advanced glycation end products, with O1 and O2 exhibiting the most pronounced effects. Mechanistically, these pigments covalently modified amino acid residues such as lysine, glutamine, and cysteine on BSA, thereby reducing the number of potential glycosylation sites. This provides novel insight into the anti-glycation potential of MPs. In a complementary study, Chen et al. [12] also employed BSA as a carrier to evaluate the antioxidant capacities and binding mechanisms of four pigment monomers: AK, MB, MS, and RP. Among them, MB exhibited the strongest antioxidant activity, as evidenced by DPPH radical scavenging capacity and total antioxidant activity assays. Furthermore, MB and RP displayed stronger binding affinities to BSA compared with AK and MS, supporting the notion that MPs not only function as natural colorants but may also serve as promising dietary supplements for applications in the food industry.

Furthermore, Alqurashy et al. [13] identified *Monascus* red pigment as a potential hepatoprotective agent. In a model of liver dysfunction induced by hydroxyapatite nanoparticles (HANP), administration of red pigment significantly alleviated hepatic injury. The protective effects were associated with attenuation of HANP-induced oxidative stress, achieved through restoration of antioxidant defenses, inhibition of lipid peroxidation, and regulation of pro-oxidative markers. In addition, *Monascus* red pigment enhanced mitochondrial function and energy metabolism by upregulating the expression of key metabolic regulators, including AMP-activated protein kinase (AMPK), PPAR γ coactivator-1α, and mitochondrial transcription factor A. These findings highlight the potential of *Monascus* red pigment as a multifunctional bioactive compound with hepatoprotective and metabolic regulatory properties.

Despite their broad spectrum of physiological activities, the wider industrial application of MPs remains constrained by several challenges, including limited stability under certain processing conditions, poor water solubility of specific pigment classes, and the frequent co-production of the nephrotoxic mycotoxin citrinin. Overcoming these limitations through fermentation process optimization and metabolic engineering is crucial for the safe, stable, and sustainable use of MPs in the food, pharmaceutical, and cosmetics industries.

### 3.3. GABA

In addition to pigments and MK, *Monascus* species are also capable of producing GABA, a non-protein amino acid with well-documented physiological functions, including neurotransmission, hypotensive activity, and regulation of glucose and lipid metabolism. GABA production in *Monascus* is primarily mediated by the enzyme glutamate decarboxylase, which catalyzes the irreversible decarboxylation of L-glutamate to GABA [62]. Its yield is influenced by multiple factors, including fermentation conditions, substrate composition, and fermentation duration. Wu et al. [14] developed an optimized SSF strategy to enhance GABA accumulation in RYR. Under optimal conditions—using brown rice and bran in a 9:1 (*w*/*w*) ratio, an inoculum size of 21.5 mL/100 g, a stirring frequency of once every 9 h, and an incubation period of 7.2 days-the resulting starter culture exhibited a viable spore count of 4.15 × 10^7^ spores/g, α-amylase activity of 155 U/g, and saccharifying capacity of 3260 U/g. These parameters enabled *Monascus purpureus* M162 to produce 15.93 mg/g of GABA, representing a 150–267% increase compared with the parental strain.

The biological functions of GABA have been widely documented. As a neurotrophic compound, GABA has been incorporated into dietary supplements for patients with depression and other psychological disorders [63]. In metabolic regulation, GABA improves insulin sensitivity in individuals with type 2 diabetes, while in diabetic mouse models it has been shown to reduce fat and kidney weight, increase liver weight, regulate serum lipid levels, and enhance glucose tolerance [64]. GABA has also demonstrated anti-fatigue properties by lowering serum concentrations of blood lactic acid, blood urea nitrogen, and lactate dehydrogenase, while simultaneously increasing glycogen and ATP content in liver and muscle tissues [65]. Moreover, as observed solely in in vitro studies, GABA exerts anticancer activity by inducing apoptosis and inhibiting proliferation and metastasis, as evidenced by its ability to suppress the growth of Molt-4 and L1210 leukemia cells and inhibit both the growth and dissemination of colon cancer cells [66,67].

Despite these diverse bioactivities, the health evaluation of *Monascus*-derived GABA remains limited, largely due to substantial variability in yield among strains and the frequent co-production of undesirable metabolites such as citrinin. Addressing these challenges through strain improvement and metabolic engineering will be critical to realizing the full potential of *Monascus*-derived GABA as a functional food ingredient or therapeutic agent.

### 3.4. Other Metabolites

In addition to the bioactive components with recognized health benefits, *Monascus* species are also capable of producing citrinin, a mycotoxin that represents a major safety concern and a key barrier to the broader application of *Monascus*-derived products [20] (Table 3). Citrinin is a polyketide secondary metabolite with well-documented nephrotoxic, hepatotoxic, and genotoxic effects in both animal models and cellular systems, and it has been implicated in renal dysfunction and potential carcinogenicity. Its occurrence is strongly influenced by the *Monascus* strain, fermentation substrate, and environmental conditions, which complicates efforts to control citrinin contamination in industrial production [68]. Regulatory agencies worldwide have established strict limits on citrinin levels in foods and dietary supplements to ensure consumer safety. For instance, the European Union permits a maximum citrinin concentration of 2 mg/kg in commercial products [69]. To mitigate this safety risk, recent research has focused on optimizing fermentation strategies and developing strains with reduced or eliminated citrinin production. For example, Yang et al. [70] demonstrated that moderate exposure of *Monascus* cultures to a magnetic field decreased intracellular iron content, which in turn promoted pigment biosynthesis while simultaneously suppressing citrinin production. Despite these advances, achieving consistent large-scale production of pigments with minimal or no citrinin contamination remains a critical challenge for the safe commercialization of *Monascus*-derived products. In addition to the metabolites described above, *Monascus* fermentation products also yield dimerumic acid (DMA), a metabolite with notable antioxidant properties. DMA has been shown to effectively attenuate liver injury and inflammation by reducing oxidative stress in both in vitro and in vivo models. Furthermore, it can suppress reactive oxygen species-induced invasion of colorectal adenocarcinoma cells. These findings suggest that DMA represents a promising functional ingredient with potential applications in disease prevention and health promotion [71,72].

## 4. Application in Health Food Development

The integration of *Monascus*-derived ingredients into health-oriented products has expanded significantly, supported by growing evidence of their multifunctional benefits [73].

### 4.1. Functional Foods

According to one prevalent definition originating from the EU project Functional Food Science in Europe, a food is considered functional when it has been convincingly demonstrated to beneficially affect one or more target functions in the body beyond its basic nutritional value. Such effects must be relevant to improved health and well-being and/or a reduction in disease risk [74]. The U.S. Department of Agriculture defines functional foods as foods in either their natural or processed forms that contain biologically active compounds, which may be known or yet unidentified. When ingested in specific, effective, and non-toxic levels, these compounds deliver a health benefit that is clinically proven and well-documented, pertaining to the prevention, management, or treatment of chronic disease [75].

*Monascus* is incorporated into a variety of functional food matrices to enhance their health-promoting properties. In grain foods, such as soybean [76], Adlay [77], bread [78,79], noodles and rice noodles [80,81,82], it serves as a natural colorant and functional additive that improves antioxidant capacity and supports lipid metabolism. In dairy products like yogurt and cheese, *Monascus*-fermented substrates, such as RYR and durian seed, are utilized as natural colorants in yogurt, imparting a stable red hue without inhibiting lactic acid bacteria viability [83,84]. Their addition enhances antioxidant properties and total phenolic content. Pigment stability during storage and consumer acceptance are well-documented, particularly at lower concentrations or with fruit incorporation [84,85]. *Monascus* species, particularly *M. ruber* and *Monascus purpureus*, are applied in cheese to impart red pigmentation and produce functional metabolites like lovastatin, while minimizing citrinin under controlled conditions [86]. Their incorporation enhances proteolysis, modifies texture, and improves sensory attributes without bitterness or rancidity [87,88]. These fungi show great potential for developing novel functional cheeses with health benefits and consumer appeal. 

*Monascus* species, particularly *Monascus purpureus*, are applied in Pu-erh tea fermentation to modify its flavor profile and enhance health-related properties. *Monascus purpureus* has established itself as a pivotal functional starter culture for enhanced pile fermentation of ripe Pu-erh tea (RIPT), with definitive regulatory effects on the dynamic formation of the tea’s characteristic flavor profile. Inoculation with *Monascus purpureus* across six pile-turning stages of 44-day fermentation drives a fundamental shift in volatile organic compounds, transforming the floral and fruity notes of sun-dried green tea (dominated by terpenes like linalool and β-myrcene) into RIPT’s iconic aged aroma, with the sixth pile-turning stage identified as the critical window for signature flavor maturation. *Monascus purpureus* mediates microbial methylation of phenolic acids, drastically elevating the content and odor activity values (OAVs) of methoxybenzene derivatives, including 1,2-dimethoxybenzene, 1,2,3-trimethoxybenzene, 1,2,4-trimethoxybenzene, and 1,2,3,4-tetramethoxybenzene. Among these, 1,2,3-trimethoxybenzene shows the most dramatic OAV change, acting as the core contributor to RIPT’s distinctive stale-Qu aroma. Additionally, *Monascus purpureus* modulates secondary flavor pathways: enhancing β-carotene degradation to produce floral norisoprenoids, promoting phenylalanine metabolism to generate phenylethanol and vanillin, and facilitating lipid oxidation to yield subtle mushroom-like and waxy notes, enriching flavor complexity. Molecular docking confirms these methoxybenzenes bind strongly to human olfactory receptors via hydrogen bonds and hydrophobic interactions, explaining their prominent sensory performance. Collectively, *Monascus purpureus* enables targeted regulation of RIPT flavor formation, supporting quality control and standardized production of fermented Pu-erh tea [89], and significantly increases theabrownins and lovastatin [90]. These changes contribute to improved lipid-lowering, anti-inflammatory, and anti-atherosclerotic effects compared to non-inoculated tea [90,91]. Such findings highlight the potential of *Monascus* in developing value-added fermented teas with desired sensory and functional attributes. These applications capitalize on the public preference for natural ingredients and fortified foods that offer preventive health benefits beyond basic nutrition.

The retention of structural integrity and biological activity of RYR bioactive metabolites during food manufacturing and storage is a fundamental prerequisite for the consistent efficacy, label compliance, and regulatory qualification of RYR-fortified products. Unlike purified pharmaceutical statins, RYR metabolites are incorporated into complex food matrices, where their stability is governed by intrinsic molecular properties, matrix characteristics, and industrial processing conditions, with direct implications for the product heterogeneity and safety uncertainties detailed throughout this review.

### 4.2. Dietary Supplements 

Dietary supplements, also referred to as nutraceuticals, are manufactured products derived from foods, isolated nutrients, or food-based substances. They are commonly formulated in pharmaceutical forms, such as tablets, powders, and liquid extracts—presentations that are not typically recognized as conventional food items [92]. RYR, also known as Xuezhikang, Cholestin, Hypocol, or Zhitai, has been used in traditional Chinese medicine to regulate blood lipids and circulation [93]. It has been widely used for its potential efficiency on inflammation, blood pressure, blood glucose, cancer, and osteoporosis [94]. RYR is recognized as a food supplement in Europe, in addition to being consumed in Asian countries. It is important to note that RYR does not have approval as a food supplement in Switzerland. Combining RYR with other plant extracts continues to look promising. When bergamot and artichoke are added, the mixture may act synergistically against high cholesterol [95]. Cicero et al. [6] observed an 18.2% drop in LDL-C after patients took RYR plus artichoke and banaba extracts for six weeks. Yet Gerards et al. [8] warned in 2015 that trial heterogeneity is large and that RYR often beats only a weak control, whereas standard statin trials still match or surpass these outcomes. Peng et al. [96] concluded in 2017 that RYR is a useful lipid-lowering option, especially for statin-intolerant individuals. In 2020, Iskandar et al. [97] tested the branded product NutraforChol^®^ in a double-blind, randomized, placebo-controlled design and recorded a 15% fall in total cholesterol and a 20% fall in LDL-C versus placebo. Across the studies, patients generally followed the prescribed doses. Although the cholesterol-lowering power of RYR is no longer disputed, the exact content of active monacolins differs among products, and the overall expense is similar to that of conventional statin therapy.

Two studies systematically characterized the profile, content, and heterogeneity of active monacolins in distinct categories of RYR samples. Avula et al. [18] established a validated UHPLC-QToF-MS method for the quantification of primary active monacolins in authentic RYR, commercial raw materials, and dietary supplements. Righetti et al. [19] applied a UHPLC-TWIMS-QTOF metabolomic strategy to profile 36 active monacolin congeners in commercial RYR food supplements. The differences in active monacolins across various RYR sample types are summarized in Table 4.

### 4.3. Traditional Fermented Foods

Beyond modern applications, *Monascus* continues to play a vital role in traditional fermented foods across Asia. RYR, a product of *Monascus purpureus.*, serves as a versatile functional starter and bioactive enhancer in various fermented foods. In *Monascus* vinegar (MV) brewing, RYR provides endogenous amylases and secondary metabolites (e.g., MPs, MK, GABA) that facilitate starch hydrolysis, alcoholic fermentation, and acetic acid bioconversion, thereby endowing MV with superior antioxidant, anti-inflammatory, and lipid-lowering properties compared to common vinegars [98,99,100,101]. RYR-derived melanoidins further contribute to health benefits by scavenging reactive oxygen species, protecting the intestinal barrier, modulating gut microbiota, and alleviating high-fat-diet-induced liver inflammation [101,102]. Similarly, in tofuyo (red yeast fermented tofu), RYR supplies hydrolytic enzymes such as aspartic proteases and carboxypeptidases, which break down soybean proteins into peptides and free amino acids-improving texture, enhancing umami flavor, and generating bioactive compounds like ACE-inhibitory peptides and GABA for potential antihypertensive effects [98,99]. In Hongqu rice wine, RYR is incorporated across saccharification, fermentation, and aging stages, where it secretes α-amylase and glucoamylase to boost sugar and ethanol production, releases pigments and lovastatin to inhibit contaminants, and promotes the formation of melanoidins and flavor compounds (e.g., acetoin and tetramethylpyrazine) that enhance antioxidant activity and nutty/creamy flavors [98,99]. Additionally, in soy sauce processing, *Monascus*-fermented rice elevates levels of MK and GABA, increases amino nitrogen and glutamic acid for umami taste, improves color and antioxidant capacity, and complies with safety standards by not introducing detectable citrinin [3,103]. Thus, across multiple applications, RYR acts as a natural multi-functional agent that boosts fermentation efficiency, improves sensory qualities, and enhances health-related functionalities. These traditional products are now being reevaluated through a functional food lens, with research highlighting their potential in microbiota modulation and metabolic health, thereby bridging cultural heritage and contemporary health science.

## 5. Safety Considerations and Regulatory Frameworks

### 5.1. Safety, Toxicology, and the Global Regulatory Landscape

The unique position of RYR at the interface of food and medicine necessitates a rigorous evaluation of its safety. The discussion around RYR safety is twofold, encompassing both the potential for contamination with microbial toxins and the inherent pharmacological risks associated with its primary active ingredient. This dual concern is reflected in a complex and highly fragmented global regulatory landscape.

### 5.2. Toxicological Assessment: The Citrinin Challenge and Detection Methodologies

The most significant toxicological concern associated with RYR production is the potential for contamination with citrinin. Citrinin is a mycotoxin known to be nephrotoxic (damaging to the kidneys) that can be produced by some strains of *Monascus*, as well as other fungi like *Penicillium* and *Aspergillus* [104,105]. The production of citrinin is strongly influenced by the specific fungal strain employed and the conditions under which fermentation is carried out. Due to the potential health risks associated with citrinin contamination, regulatory authorities—particularly in the European Union—have established stringent maximum limits for citrinin in RYR-based dietary supplements. For instance, the European Commission has set a maximum permitted level of 100 µg/kg. To comply with these regulations and ensure product safety, manufacturers must adopt rigorous practices, including the careful selection of fungal strains that do not produce citrinin, along with tightly controlled fermentation processes. Conventional methods for citrinin analysis include thin-layer chromatography with fluorescence detection, often enhanced by aluminum chloride or other derivatization agents. However, HPLC coupled with fluorescence detection remains the most widely applied technique, offering improved accuracy and sensitivity, with typical excitation/emission wavelengths around 330–340 nm and 495–512 nm. For confirmatory and multi-mycotoxin analysis, LC-MS is increasingly employed, enabling precise quantification even at trace levels. In addition, screening methods such as ELISA and lateral flow tests provide rapid, on-site alternatives, though they may require validation against chromatographic techniques. The choice of method often depends on the required sensitivity, specificity, and throughput [106].

### 5.3. Clinical Safety Profile and Statin-Associated Side Effects

Given that MK is structurally identical to lovastatin, RYR presents a risk profile similar to that of statin medications. Commonly reported adverse effects include myopathy, which manifests as muscle pain, tenderness, or weakness, as well as headache and gastrointestinal symptoms such as heartburn and flatulence [11]. Although clinical trials generally indicate that RYR is well tolerated with a low frequency of adverse events, the potential for more severe complications, including rhabdomyolysis and hepatotoxicity, albeit rare, cannot be dismissed [11]. Consumer forums and case reports further underscore these concerns, noting that individuals with a history of statin intolerance often express similar apprehensions regarding the use of RYR [107]. Consequently, it is advisable for individuals-particularly those using higher doses-to take RYR under medical supervision. Caution is also warranted for those with pre-existing liver conditions or individuals concurrently taking medications metabolized by the same hepatic enzymes.

### 5.4. Standardization and International Regulatory Divergence

The food–drug paradox of RYR is most evident in its fractured and inconsistent regulatory status across the globe. Different countries and regions have adopted vastly different approaches to classifying, limiting active ingredients, controlling contaminants, and approving health claims for RYR products, creating significant confusion for manufacturers, clinicians, and consumers worldwide (Table 5).

In the United States, the FDA considers RYR products that contain more than trace amounts of MK to be unapproved new drugs, as lovastatin is an approved drug active ingredient. Consequently, supplements are legally prohibited from being marketed based on a specified MK content. This has led to a market where products can vary widely in potency, but manufacturers cannot provide consumers with clear information about the dose of the active compound.

The 2025 scientific opinion from the European Food Safety Authority Panel on Nutrition, Novel Foods and Food Allergens reaffirms that systemic exposure to MK derived from RYR, even at daily intake levels as low as 3 mg, is associated with severe adverse events—most notably rhabdomyolysis and other musculoskeletal toxicities, as well as clinically significant hepatic injury. Stakeholder-submitted supplementary data throughout the EU regulatory scrutiny period were insufficient to establish the safety of MK-containing RYR food supplements at doses below 3 mg/day, nor to define a tolerable daily intake threshold without safety concerns for either the general population or vulnerable subpopulations.

Compounding these safety uncertainties, commercially available RYR supplements exhibit extreme inter-product heterogeneity in total MK content and the ratio of lactone form to bioactive hydroxy acid-form MK, with widespread discrepancies between analytically measured values and product label declarations. Consistent with EU regulatory frameworks, RYR supplements are contraindicated for pregnant and lactating individuals, persons under 18 or over 70 years of age, and those receiving concomitant statin therapy. Furthermore, the majority of available RYR clinical trials are statistically underpowered to detect long-term toxicities or rare adverse events, mandating rigorous caution regarding the unsupervised, over-the-counter use of RYR-containing supplements.

A fundamental barrier to the responsible clinical and consumer use of RYR is posed by the absence of a harmonized international standard governing its potency, purity, and safety. Systemic uncertainty for all stakeholders is created by cross-jurisdictional fragmentation of regulatory frameworks, analytical methodologies, and safety assessment criteria, with tangible public health impacts corroborated by global post-marketing surveillance data.

First and foremost, irreconcilable variability in active moiety quantification—the cornerstone of consistent, safe dosing—is driven by divergent potency specifications. Total RYR monacolins are restricted to <3 mg per daily serving under EU Commission Regulation 2022/860 [108], with no mandatory method to distinguish active hydroxy acid-form MK from its inactive lactone form. China’s GB 1886.19-2015 [109] defines potency exclusively as total MK, mandating a 0.2% *w*/*w* minimum content and 1–4 mg daily recommended intake, while the US FDA classifies MK-containing RYR as an unapproved new drug with no standardized potency definition. 2025 EFSA data show commercial RYR total MK content ranges 0.45–7.84% *w*/*w*, with label deviations of−37% to +266% and lactone-to-hydroxy acid ratios varying 1:1 to 114:1. This phenomenon indicates that consistent dosing cannot be guaranteed even with strict label adherence.

Compounding these challenges, critical safety gaps are left by misaligned purity and contaminant controls. For nephrotoxic citrinin, a 100 μg/kg limit is enforced under EU Regulation 2023/915 [110], a 50 μg/kg limit in China’s QB/T 2847-2023 [111], and only non-binding FDA guidance is issued. No global mandate is established for synthetic statin adulteration screening or cytotoxic metabolite limits.

**Table 5:** Comparison of Regulatory Frameworks for RAR in Major Markets.

Country/Region	Regulatory Classification	Permissible Monacolin K (MK) Daily Intake Limit	Maximum Limit of Citrinin	Permitted Health Claims
United States	Dietary supplement; products with more than trace amounts of MK are classified as unapproved new drugs (2013) [17]	Not specified; no legal allowable limit for MK in dietary supplements (2013) [17]	No mandatory federal limit; only non-binding guidance for supplements (2013) [17]	General structure/function claims allowed; explicit disease risk reduction claims (e.g., “lowers cholesterol”) are prohibited (2013) [17]
European Union (EU)	Food supplement (2022) [108]	<3 mg total monacolins per daily serving (Commission Regulation 2022/860) (2022) [108]	100 μg/kg (Commission Regulation 2023/915) (2023) [110]	Approved EFSA health claim: “Monacolin K from RYR contributes to the maintenance of normal blood cholesterol levels” (for daily doses ≥10 mg MK, subject to the 3 mg serving limit) (2024) [7]
China	Dual classification: 1) Health food (blue hat certification); 2) Prescription drug (e.g., Xuezhikang)(2024) [49]	Health food: 1~4 mg MK per daily recommended intake; Prescription drug: 10 mg MK per daily standard dose (2024) [49]	50 μg/kg (QB/T 2847-2023) [111]	Approved specific health function claims (e.g., “assists in lowering blood lipids”) for registered health foods; drug indications for prescription products (2024) [51]
Japan	Traditional food; post-2024 Beni-Koji crisis: designated as food with special health monitoring requirements (2021) [3]	No mandatory legal limit pre-2024; post-crisis provisional guideline: <3 mg MK per daily serving (2021) [3]	No universal mandatory limit pre-2024; post-crisis provisional limit: 100 μg/kg (2021) [3]	No official approved health claims for MK-containing RYR; only general functional food labeling allowed (2021) [3]
Republic of Korea	Health functional food (HFF) under MFDS regulation (2021) [3]	≤4 mg MK per daily recommended intake (2021) [3]	50 μg/kg (2022) [20]	Approved health claim: “Helps maintain normal blood cholesterol levels” for registered HFF products (2021) [3]
Australia and New Zealand	Complementary medicine (Australia); dietary supplement (New Zealand) (2024) [112]	≤3 mg MK per daily serving (2024) [112]	100 μg/kg (aligned with EU limits) (2024) [112]	Permitted low-level cholesterol maintenance claims; therapeutic claims require medicine registration (2023) [9]
Canada	Natural health product (NHP) under Health Canada regulation (2024) [7]	≤10 mg MK per daily serving (must be labeled as lovastatin equivalent) (2024) [7]	100 μg/kg (2024) [7]	Approved claim: “Helps maintain/support healthy cholesterol levels” for licensed NHPs (2024) [7]

Relatedly, evidence-based use is undermined by discordant safety frameworks. EFSA (2018, 2025) [31,32] concluded no tolerable daily MK intake exists, with severe adverse events at doses as low as 3 mg/day, prompting EU mandatory contraindication labeling; far weaker or no such rules are enforced in China and the US. These gaps were starkly illustrated by the 2024 Japanese Beni-Koji crisis, where >1000 renal impairment cases and more than 20 deaths were linked to contaminated RYR.

Collectively, as detailed in Table 5, the divergent regulatory frameworks across major global markets have created fundamental barriers to the standardized development of RYR products. Responsible RYR use will remain compromised without a unified international standard harmonizing potency analytics, contaminant limits, and safety protocols, perpetuating product heterogeneity and preventable consumer harm.

## 6. Current Challenges and Future Perspectives

The complexity of RYR introduces a set of substantial challenges that currently constrain its broader application and pose potential risks for consumers. The most critical of these challenges can be summarized as follows:

Lack of Standardization: The profound variability in the bioactive content (especially MK) and the potential for citrinin contamination across commercial products is the single greatest issue. This inconsistency, driven by differences in fungal strains and fermentation processes, makes it difficult for consumers and clinicians to select an effective and safe product.

Further Clinical Validation Required: Although the efficacy of lipid-lowering is well-established, the potential health advantages of RYR beyond this scope, such as its impact on metabolic syndrome, gut health, and inflammation, rely mainly on preclinical evidence. It is imperative to conduct rigorous and well-structured human clinical trials promptly to substantiate these assertions.

Regulatory Inconsistency: The fragmented and contradictory global regulatory environment creates confusion, hinders international trade, and fails to adequately protect consumers while ensuring access to beneficial products.

Addressing these unresolved challenges provides critical, evidence-based directions for future research and development. Investigation into RYR remains far from complete; further advancement in this field will be underpinned by advances in modern biotechnology and a more nuanced understanding of its nutritional mechanisms, alongside rigorous resolution of persistent citrinin control, regulatory compliance, and long-term human safety gaps at clinically effective doses.

Enhancing bioavailability and drug delivery systems: Investigating advanced formulations and drug delivery techniques presents significant potential for improving the therapeutic efficacy of RYR. Approaches such as microencapsulation, nanoemulsions, and solid dispersion systems can safeguard sensitive bioactive components from degradation within the gastrointestinal tract while simultaneously increasing their solubility and bioavailability. This progress may facilitate the administration of lower yet more effective doses, thereby potentially reducing the risk of adverse reactions.

Precision Nutrition: As the comprehension of the RYR metabolome advances, it may soon be feasible to transcend the conventional standardized approach. Future advancements could enable the design of well-defined, specialized RYR formulations tailored for precision nutrition. For instance, a preparation enriched in MK could be utilized for targeted cholesterol management, whereas another formulation abundant in bioactive pigments and GABA might be developed for individuals seeking comprehensive cardiovascular support, with particular emphasis on blood pressure regulation and anti-inflammatory benefits [113].

## 7. Conclusions

RYR represents a compelling instance of a traditional remedy whose efficacy has been substantiated through modern scientific inquiry. Its evolution from a staple in ancient diets to a globally utilized nutraceutical underscores its potent bioactive properties—most notably, a clinically demonstrated capacity to manage hyperlipidemia. This review has consolidated extensive evidence indicating that the health benefits of RYR stem from its complex metabolome: the HMG-CoA reductase inhibitor MK acts as the principal bioactive component within this metabolome, while its effects are significantly augmented by a suite of complementary constituents such as antioxidant and anti-inflammatory pigments, GABA, and other bioactive compounds. Furthermore, adverse effects associated with statins include myopathy, headache, and gastrointestinal inflammation.

In summary, the multifaceted trajectory of RYR—spanning its origins in traditional medicine, its pharmacological validation, and its current role in regulatory discourse—serves as an instructive paradigm for the broader functional food and nutraceutical sector. The insights gained from its development highlight the critical importance of standardization, the need for comprehensive safety evidence, the inevitability of discussions regarding the food–drug boundary, and the potential applications of metabolic engineering. Collectively, these insights provide a strategic framework and a prudent caution for the future advancement of evidence-based natural health products.

## Figures and Tables

**Table 2:** Different Engineering strategies in *Monascus*.

No.	Engineering Strategy	Target Gene/Element	Key Outcomes	Reference
1	CRISPR/Cas9 editing; Overexpression	*CtnC* (MFS transporter)	Citrinin ↓12.93–67.34%; Red pigment ↑228.70%	[39]
2	CRISPR/Cas9 editing; Overexpression	*CtnD* (oxidoreductase)	Citrinin ↓ >98% (editing); Citrinin ↑ 31.7–82.1% (overexpression)	[38]
3	CRISPR/Cas9 editing; Overexpression	*comp54181_c0* (transcription factor)	MK ↑ 11.9%; Pigments ↑ 20–48% (knockout)	[35]
4	CRISPR/Cas9 editing; Complementation	*rtt109* (histone acetyltransferase)	Pigments ↑ 37.43–42.32%; Citrinin ↑ 34.54%	[36]
5	CRISPR/Cas9 (dual sgRNAs)	*MpigI*/*MpigI′* (negative regulators)	Pigments ↑ 2.5-18.5-fold; No citrinin detected	[40]
6	MIDBE base editor	Random genomic loci	MK ↑ 977.1%	[41]
7	CRISPR/Cas9 editing; Overexpression	*ctnA* (transcriptional activator)	Citrinin ↓ 78–83% (knockout); Citrinin ↑ 20–500% (overexpression)	[37]

The symbol “↓” indicates a decrease, and “↑” indicates an increase.

**Table 3:** Major Bioactive Compounds Identified in RYR.

Compound Class	Specific Compound	Chemical Structure	Primary Reported Bioactivity
Monacolins	Monacolin K	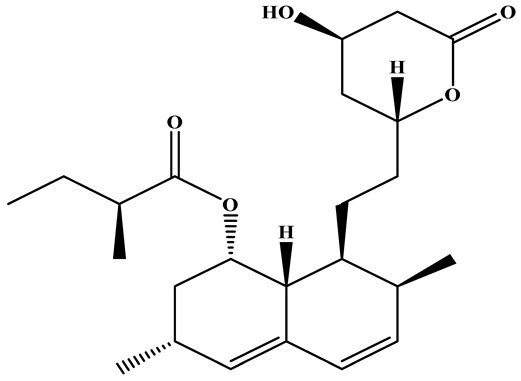	HMG-CoA reductase inhibition; Cholesterol-lowering
	Dihydromonacolin K	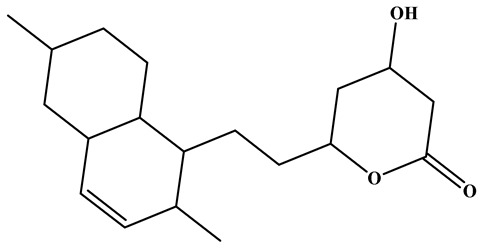	HMG-CoA reductase inhibition
Pigments (Yellow)	Monascin	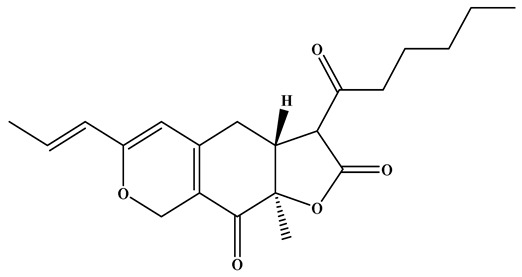	Antioxidant; Anti-inflammatory; Anti-cancer
	Ankaflavin	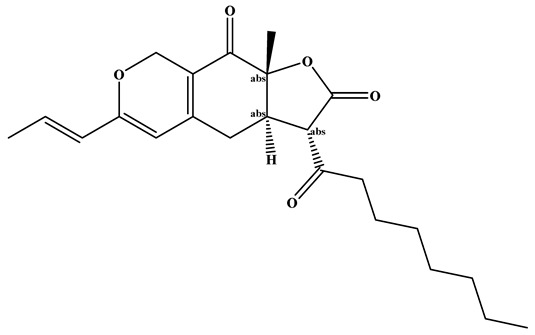	Antioxidant; Anti-inflammatory; Anti-cancer
Pigments (Orange)	Rubropunctatin	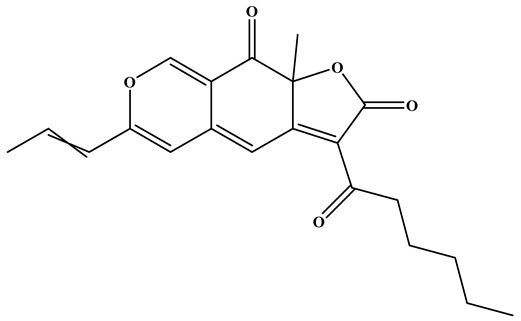	Antioxidant; Anti-inflammatory
	Monascorubrin	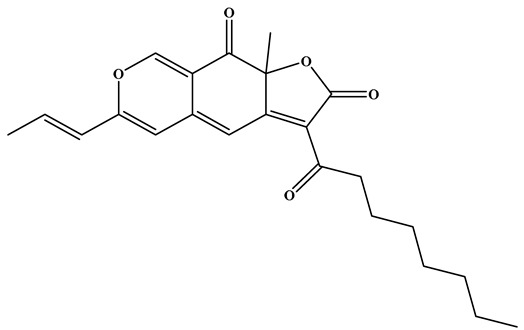	Antioxidant; Anti-inflammatory
Pigments (Red)	Rubropunctamine	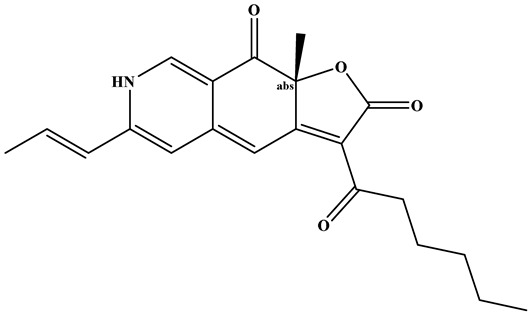	Antioxidant; Water-soluble colorant
	Monascorubramine	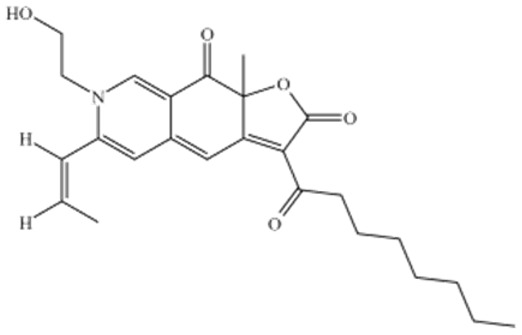	Antioxidant; Water-soluble colorant
Amino Acids	γ-Aminobutyric Acid (GABA)	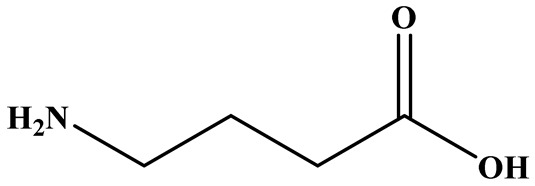	Neurotransmitter; Hypotensive; Anti-inflammatory
Sterols	Ergosterol	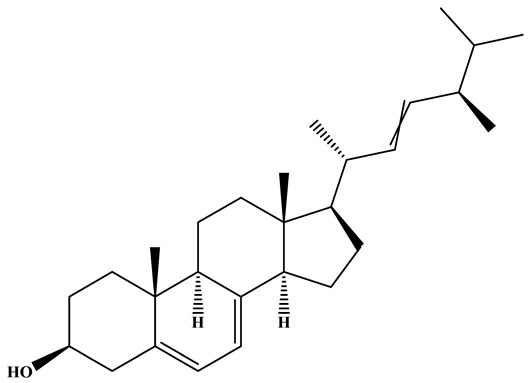	Provitamin D2; Cholesterol-lowering potential
	β-Sitosterol	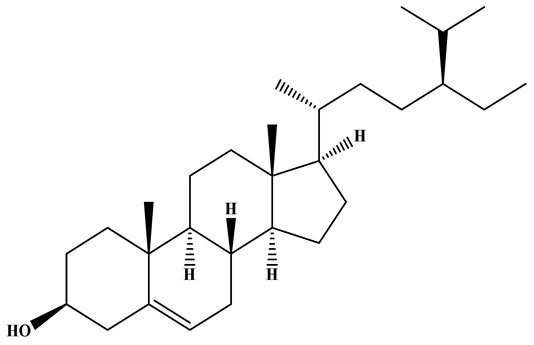	Inhibits dietary cholesterol absorption
Polysaccharides	*Monascus* Polysaccharides	Complex polymers	Immunomodulatory; Prebiotic potential

**Table 4:** Differences in active monacolins among different RYR sample categories.

Sample Category	Primary Active Monacolin (MK) Content Range	MK:MKA Ratio (Characteristic Range)	Secondary Active Monacolins Profile and Content
Authentic RYR samples	1.9–2.3 mg/g dry weight; 1.2–1.38 mg per 600 mg RYR	1.4:1 to 1.6:1	Dehydro-MK detected at 0.05 mg per 600 mg RYR; Monacolin J and compactin were DUL; no other monacolins at quantifiable levels
Commercial RYR raw materials	ND (10 samples); 0.7–24.3 mg/g dry weight (21 samples with quantifiable monacolins)	0.5:1 to 7.5:1 (for samples with detectable MKA)	Dehydro-MK ranged from DUL to 2.15 mg/g dry weight; compactin ranged from DUL to 0.81 mg/g dry weight; Monacolin J was mostly DUL
RYR dietary supplements	0.03–2.18 mg per 600 mg labeled RYR (40-fold variation); 0.22–5.23 mg per 1200 mg labeled RYR (20-fold variation); 0.12–10.46 mg daily intake per label recommendation	1.1:1 to 4.9:1 (8 samples with detectable MKA); MKA was ND in 6 samples	Dehydro-MK ranged from 0.01 to 1.46 mg per 600 mg RYR; compactin ranged from DUL to 1.46 mg per dosage unit; Monacolin J was mostly DUL
Pure RYR dietary supplements	MK accounted for 22–60% of total monacolins (mean 33%); MK + MKA accounted for 27–81% of total monacolins (mean 46%)	Not explicitly reported, with high inter-sample variation	36 monacolins detected in total; minor monacolins accounted for 8–54% of total monacolins + pigments (mean 37%); Dehydro-MK 1–20% of total monacolins (mean 13%); Dihydro-MK 1–14% of total monacolins (mean 9%)
Multi-ingredient RYR dietary supplements	MK accounted for 22–60% of total monacolins (mean 33%); MK + MKA accounted for 27–81% of total monacolins (mean 46%)	Not explicitly reported, with high inter-sample variation	36 monacolins detected in total; minor monacolins accounted for 8–54% of total monacolins + pigments (mean 37%); 26/26 samples had dehydro- + dihydro-MK >20% of total monacolins (range 7–43%)

## Data Availability

The original contributions presented in the study are included in the article. Further inquiries can be directed to the corresponding authors.

## Abbreviations

The following abbreviations are used in this manuscript:

RYR	Red yeast rice
MK	Monacolin K
GABA	γ-aminobutyric acid
SSF	Solid-state fermentation
LDL-C	Low-density lipoprotein cholesterol
TC	total cholesterol
MPs	*Monascus* pigments
SmF	Submerged fermentation
ND	not detected
EFSA	European Food Safety Authority
HMG-CoA	Hydroxy methylglutaryl coenzyme A
HDL-C	High-density lipoprotein cholesterol
MS	Monascin
AK	Ankaflavin
O1	Rubropunctatin
O2	Monascorubrin
RP	Rubropunctamine
MB	Monascorubramine
PPARγ	Proliferator-activated receptor γ
BSA	Bovine serum albumin
RB	rubropunctatin
HANP	Hydroxyapatite nanoparticles
AMPK	Activated protein kinase
DMA	Dimerumic acid
OAVs	odor activity values
MV	*Monascus* vinegar

## References

[1] Zhu B., Qi F., Wu J., Yin G., Hua J., Zhang Q., Qin L. (2019). Red Yeast Rice: A Systematic Review of the Traditional Uses, Chemistry, Pharmacology, and Quality Control of an Important Chinese Folk Medicine. Front. Pharmacol..

[2] Liu S., Xu Y., Xie J., Hu J., Wang Y., Zhang J., Lee M., Hu H., Ang L., Ji Z. (2025). The pharmacology and mechanism of action of *Monascus purpureus* Went: A scoping review. Front. Pharmacol..

[3] Fukami H., Higa Y., Hisano T., Asano K., Hirata T., Nishibe S. (2021). A Review of Red Yeast Rice, a Traditional Fermented Food in Japan and East Asia: Its Characteristic Ingredients and Application in the Maintenance and Improvement of Health in Lipid Metabolism and the Circulatory System. Molecules.

[4] Nguyen T., Karl M., Santini A. (2017). Red Yeast Rice. Foods.

[5] Ma J., Li Y., Ye Q., Li J., Hua Y., Ju D., Zhang D., Cooper R., Chang M. (2000). Constituents of Red Yeast Rice, a traditional Chinese food and medicine. J. Agric. Food Chem..

[6] Cicero A.F.G., Fogacci F., Banach M. (2019). Red Yeast Rice for Hypercholesterolemia. Methodist DeBakey Cardiovasc. J..

[7] Trogkanis E., Karalexi M.A., Sergentanis T.N., Kornarou E., Vassilakou T. (2024). Safety and Efficacy of the Consumption of the Nutraceutical “Red Yeast Rice Extract” for the Reduction of Hypercholesterolemia in Humans: A Systematic Review and Meta-Analysis. Nutrients.

[8] Gerards M.C., Terlou R.J., Yu H., Koks C.H.W., Gerdes V.E.A. (2015). Traditional Chinese lipid-lowering agent Red Yeast Rice results in significant LDL reduction but safety is uncertain—A systematic review and meta-analysis. Atherosclerosis.

[9] Cicero A.F.G., Fogacci F., Stoian A.P., Toth P.P. (2023). Red Yeast Rice for the Improvement of Lipid Profiles in Mild-to-Moderate Hypercholesterolemia: A Narrative Review. Nutrients.

[10] Liasi E., Kantilafti M., Hadjimbei E., Chrysostomou S. (2024). Monacolin K supplementation in patients with hypercholesterolemia: A systematic review of clinical trials. Semergen.

[11] Feng Y., Shao Y., Chen F. (2012). *Monascus* Pigments. Appl. Microbiol. Biotechnol..

[12] Chen Q., Wang S., Zhou J., Shen W., Jia X., Xiang L., Chen X. (2025). Analysis on the antioxidant capacities of four *Monascus* pigment components and their binding mechanisms with bovine serum albumin. LWT-Food Sci. Technol..

[13] Alqurashy N.N., Yousef M.I., El Tabakh M.A.M., Hussein A.A., Kamel M.A., El-Wakil A. (2025). Protective role of *Monascus* red pigment against hydroxyapatite nanoparticle-induced liver injury in rats via modulation of metabolic regulators. Biochem. Biophys. Res. Commun..

[14] Wu A., Li L., Zhang S., Lin Q., Liu J. (2023). Optimization of the hongqu starter preparation process for the manufacturing of red mold rice with high gamma-aminobutyric acid production by solid-state fermentation. Appl. Biochem. Biotechnol..

[15] Li S., Cai Q., Liu Q., Gong Y., Zhao D. (2024). Effective enhancement of the ability of *Monascus pilosus* to produce lipid-lowering compound Monacolin K via perturbation of metabolic flux and histone acetylation modification. Food Res. Int..

[16] Zhou W., Guo R., Guo W., Hong J., Li L., Ni L., Sun J., Liu B., Rao P., Lv X. (2019). *Monascus* yellow, red and orange pigments from Red Yeast Rice ameliorate lipid metabolic disorders and gut microbiota dysbiosis in Wistar rats fed on a high-fat diet. Food Funct..

[17] Childress L., Gay A., Zargar A., Ito M.K. (2013). Review of Red Yeast Rice content and current Food and Drug Administration oversight. J. Clin. Lipidol..

[18] Avula B., Cohen P.A., Wang Y.-H., Sagi S., Feng W. (2014). Chemical profiling and quantification of monacolins and citrinin in red yeast rice commercial raw materials and dietary supplements using liquid chromatography-accurate QToF mass spectrometry: Chemometrics application. J. Pharm. Biomed. Anal..

[19] Righetti L., Dellafiora L., Rolli E., Dall’Asta C., Bruni R. (2022). Standardization issues in botanicals: A metabolomic and in silico approach to *Monascus purpureus* food supplements. Food Biosci..

[20] Kamle M., Mahato D.K., Gupta A., Pandhi S., Sharma N., Sharma B., Mishra S., Arora S., Selvakumar R., Saurabh V. (2022). Citrinin Mycotoxin Contamination in Food and Feed: Impact on Agriculture, Human Health, and Detection and Management Strategies. Toxins.

[21] Gordon R.Y., Cooperman T., Obermeyer W., Becker D.J. (2010). Marked Variability of Monacolin Levels in Commercial Red Yeast Rice Products. Arch. Intern. Med..

[22] Bule M., Khan F., Niaz K. (2019). Red Yeast Rice (*Monascus purpureus*). Nonvitamin and Nonmineral Nutritional Supplements.

[23] Musselman M.E., Pettit R.S., Derenski K.L. (2011). A Review and Update of Red Yeast Rice. J. Evid.-Based Complement. Altern. Med..

[24] Singh R., Kumar M., Mittal A., Mehta P.K. (2017). Microbial metabolites in nutrition, healthcare and agriculture. 3 Biotech.

[25] Bérdy J. (2005). Bioactive Microbial Metabolites. J. Antibiot..

[26] Wang Y., Ye F., Zhou B., Liang Y., Lin Q. (2023). Comparative analysis of different rice substrates for solid-state fermentation by a citrinin-free *Monascus purpureus* mutant strain with high pigment production. Food Biosci..

[27] Yuan X., Gao S., Tan Y., Cao J., Yang S. (2023). Production of red yeast rice rich in monacolin K by variable temperature solid fermentation of *Monascus purpureus*. RSC Adv..

[28] Guo Z., Lai Y., Gou Y., Guo J., Lian X. (2025). Screening of *Monascus* to produce high-yield monacolin K by solid-state fermentation on medium of coix seed and gluten fractions. Food Biosci..

[29] Wang Y., Gao C., Long P., Hu Z., Zhu L. (2023). Dynamic changes of active substances of rice, Pueraria and yam fermentation by *Monascus ruber*. LWT.

[30] Wu M., Wang Q., Zhang H., Pan Z., Zeng Q. (2023). Performance and mechanism of co-culture of *Monascus purpureus*, *Lacticaseibacillus casei*, and *Saccharomyces cerevisiae* to enhance lovastatin production and lipid-lowering effects. Bioprocess Biosyst. Eng..

[31] Wu Z., Zhang C., Liu Q., Zhang S., Yang Q. (2025). Combinatorial mutagenesis and fermentation optimization biotechnologies synergistically enhance monacolin K content in functional red yeast rice. Front. Microbiol..

[32] (2018). EFSA Panel on Food Additives and Nutrient Sources added to Food (ANS). Scientific opinion on the safety of monacolins in red yeast rice. EFSA J..

[33] EFSA Panel on Nutrition, Novel Foods and Food Allergens (NDA) (2025). Scientific Opinion on additional scientific data related to the safety of monacolins from red yeast rice submitted pursuant to Article 8(4) of Regulation (EC) No 1925/2006. EFSA J..

[34] The European Commission (2019). Commission Regulation (EU) 2019/1901 of 7 November 2019 amending Regulation (EC) No 1881/2006 as regards maximum levels of citrinin in food supplements based on rice fermented with red yeast *Monascus purpureus*. Off. J. Eur. Union.

[35] Zhang C., Wang H., Ablimit A., Zhao Y., Sun Q. (2025). Functional Verification of Transcription Factor *comp54181_c0* in *Monascus purpureus*. J. Basic Microbiol..

[36] Shi R., Gong P., Luo Q., Chen W., Wang C. (2023). Histone Acetyltransferase *Rtt109* Regulates Development, Morphogenesis, and Citrinin Biosynthesis in *Monascus purpureus*. J. Fungi.

[37] Zhang X., Chen W., Wang C. (2025). Regulation of citrinin biosynthesis in *Monascus purpureus*: Impacts on growth, morphology, and pigments production. Food Microbiol..

[38] Tang G., Man H., Wang J., Zou J., Zhao J. (2023). An oxidoreductase gene CtnD involved in citrinin biosynthesis in *Monascus purpureus* verified by CRISPR/Cas9 gene editing and overexpression. Mycotoxin Res..

[39] Gui Y., Tang G., Man H., Wang J., Han J. (2023). Transportation of citrinin is regulated by the CtnC gene in the medicinal fungus *Monascus purpureus*. J. Zhejiang Univ.-Sci. B (Biomed. Biotechnol.).

[40] Yoon H.R., Han S., Shin S.C., Su C.Y., Hyo J.K. (2023). Improved natural food colorant production in the filamentous fungus *Monascus ruber* using CRISPR-based engineering. Food Res. Int..

[41] Duan Y., Tan Y., Chen X., Pei X., Li M. (2023). Modular and Flexible Molecular Device for Simultaneous Cytosine and Adenine Base Editing at Random Genomic Loci in Filamentous Fungi. ACS Synth. Biol..

[42] Xiong Z., Cao X., Wen Q., Chen Z., Cheng Z. (2019). An overview of the bioactivity of monacolin K/lovastatin. Food Chem. Toxicol..

[43] Sun Q.W., Hong H.S. (2023). Research Progress on Gene Synthesis and Anticancer and Lipid-lowering Mechanism of Monacolin K. Anti-Cancer Agents Med. Chem..

[44] Zheng Y., Zheng Y., Huang Z., Zhang Y., Li J. (2025). Investigating the biosynthesis pathways and hypolipidemic mechanisms of monacolin K in *Monascus* species. Phytomedicine.

[45] Beltran D., Frutos-Lison M.D., Espin J.C., Garcia-Villalba R. (2019). Re-examining the role of the gut microbiota in the conversion of the lipid-lowering statin monacolin K (lovastatin) into its active beta-hydroxy acid metabolite. Food Funct..

[46] Martinez-Martin F., Corbella E., Sarasa I., Trias F., Petitbò D. (2022). Effects of treatment with monacolin K, berberine and coenzyme Q10 on lipid metabolism in patients with moderate cardiovascular risk. Semergen.

[47] Zhu Q., Zeng C., Peng W., Chen H., Huang H. (2025). Lovastatin alleviates DSS-induced colitis by modulating macrophage polarization via the PPARgamma-NF-kappaB pathway. Int. Immunopharmacol..

[48] De Lellis L.F., Morone M.V., Buccato D.G., Cordara M., Larsen D.S. (2024). Efficacy of Food Supplement Based on Monacolins, γ-Oryzanol, and γ-Aminobutyric Acid in Mild Dyslipidemia: A Randomized, Double-Blind, Parallel-Armed, Placebo-Controlled Clinical Trial. Nutrients.

[49] Yang C., Wu Y., Qian J., Li J. (2024). A systematic, updated review of Xuezhikang, a domestically developed lipid-lowering drug, in the application of cardiovascular diseases. Acta Pharm. Sin. B.

[50] Buzzelli L., Segreti A., Di Gioia D., Lemme E., Squeo M.R. (2024). Alternative lipid lowering strategies: State-of-the-art review of red yeast rice. Fitoterapia.

[51] Jafar M., Davood N., Ömer A., Raoofi A., Delbari A. (2022). Neuroprotective effects of Lovastatin against traumatic spinal cord injury in rats. J. Chem. Neuroanat..

[52] Chen W., Feng Y., Molnar I., Chen F. (2019). Nature and nurture: Confluence of pathway determinism with metabolic and chemical serendipity diversifies *Monascus* azaphilone pigments. Nat. Prod. Rep..

[53] Arruda G.L., Reis W.S.M., Raymundo M., Shibukawa V.P., Cruz-Santos M.M. (2025). Biotechnological potential of *Monascus*: Biological aspects, metabolites of interest, and opportunities for new products. Microbiol. Res..

[54] Gong P., Shi R., Liu Y., Luo Q., Wang C. (2023). Recent advances in *Monascus* pigments produced by *Monascus purpureus*: Biosynthesis, fermentation, function, and application. LWT-Food Sci. Technol..

[55] Chaudhary V., Katyal P., Panwar H., Kaur J., Aluko R.E. (2022). Antioxidative, anti-inflammatory, and anticancer properties of the red biopigment extract from *Monascus purpureus* (MTCC 369). J. Food Biochem..

[56] Dong C., Cheng Y., Zhang M., Chen M., Yan Z. (2024). *Monascus* pigments suppress fructose-mediated BSA glycation by trapping methylglyoxal and covalent binding to proteins. Int. J. Biol. Macromol..

[57] Lee C.L., Wen J.Y., Hsu Y.W., Pan T. (2013). *Monascus*-fermented yellow pigments monascin and ankaflavin showed antiobesity effect via the suppression of differentiation and lipogenesis in obese rats fed a high-fat diet. J. Agric. Food Chem..

[58] Wei Y., Popovich D.G. (2013). Red azaphilone pigments extracted from Red Yeast Rice induces cellular senescence and reduces viability in HepG2 cells. Biomed. Prev. Nutr..

[59] Abdollahi F., Jahadi M., Ghavami M. (2021). Thermal stability of natural pigments produced by *Monascus purpureus* in submerged fermentation. Food Sci. Nutr..

[60] Almeida A.B.d., Santos N.H., Lima T.M.D., Railany V.S., Josemar G.O.F. (2021). Pigment bioproduction by *Monascus purpureus* using corn bran, a byproduct of the corn industry. Biocatal. Agric. Biotechnol..

[61] Zheng Y., Xin Y., Shi X., Guo Y. (2010). Anti-cancer effect of rubropunctatin against human gastric carcinoma cells BGC-823. Appl. Microbiol. Biotechnol..

[62] Kusdiyantini E., Nurhayati, Ferniah R.S. (2021). Production of γ-Aminobutyric Acid (GABA) by isolated from Angkak, a mold isolated from Angkak in Semarang, Indonesia. J. Phys. Conf. Ser..

[63] Liwinski T., Lang U.E., Bruhl A.B., Schneider E. (2023). Exploring the Therapeutic Potential of Gamma-Aminobutyric Acid in Stress and Depressive Disorders through the Gut-Brain Axis. Biomedicines.

[64] Li X., Chen L., Zhu X., Lu Z., Lu Y. (2020). Effect of gamma-aminobutyric acid-rich yogurt on insulin sensitivity in a mouse model of type 2 diabetes mellitus. J. Dairy Sci..

[65] Zhang D., Xiong J., Zhao X., Gan Y. (2023). Anti-fatigue activities of γ-aminobutyric acid-enriched soymilk in an acute exercise-treated mouse model via regulating AMPK/PGC-1α pathway. Food Biosci..

[66] Mills D.J. (2021). The Aging GABAergic System and Its Nutritional Support. J. Nutr. Metab..

[67] Tufail T., Ain H.B.U., Virk M.S., Ashraf J., Ahmed Z. (2025). GABA (gamma-aminobutyric acid) enrichment and detection methods in cereals: Unlocking sustainable health benefits. Food Chem..

[68] Lin T.S., Chiu S.H., Chen C.C., Lin C.H. (2023). Investigation of monacolin K, yellow pigments, and citrinin production capabilities of *Monascus purpureus* and *Monascus ruber* (*Monascus pilosus*). J. Food Drug Anal..

[69] Kim D., Ku S. (2018). Beneficial Effects of *Monascus* sp. KCCM 10093 Pigments and Derivatives: A Mini Review. Molecules.

[70] Yang Y., Liao Q., Zhang J., Liu Y., Li L. (2026). Effect of a magnetic field on the production of *Monascus* pigments and citrinin via regulation of intracellular and extracellular iron content. Food Phys..

[71] Hong X., Deng J., Liu J., Zhong H., Ren J. (2025). Research advances in yellow pigments derived from *Monascus*, an edible filamentous fungus. Adv. Appl. Microbiol..

[72] Lee B.-H., Pan T.-M. (2013). Dimerumic acid, a novel antioxidant identified from *Monascus*-fermented products exerts chemoprotective effects: Mini review. J. Funct. Foods.

[73] Cicero A.F.G., Fogacci F., Zambon A. (2021). Red Yeast Rice for Hypercholesterolemia. J. Am. Coll. Cardiol..

[74] Diplock A.T., Aggett P.J., Ashwell M., Bornet F., Fern E.B. (1999). Scientific concepts of functional foods in Europe. Consensus document. Br. J. Nutr..

[75] Martirosyan D.M., Singh J. (2015). A new definition of functional food by FFC: What makes a new definition unique?. Funct. Foods Health Dis..

[76] Pyo Y.H., Seong K.S. (2009). Hypolipidemic effects of *Monascus*-fermented soybean extracts in rats fed a high-fat and -cholesterol diet. J. Agric. Food Chem..

[77] Shi Y.C., Liao J.W., Pan T.M. (2011). Antihypertriglyceridemia and anti-inflammatory activities of *Monascus*-fermented dioscorea in streptozotocin-induced diabetic rats. Exp. Diabetes Res..

[78] Parmigiani Monteiro A.B., Prados C.R.M.G., Silva M.D.L.R., Silva E.P., Damiani C. (2021). Production of *Monascus* pigments by solid-state cultivation of wheat grains and application in bread formulations. Int. J. Gastron. Food Sci..

[79] Liu A., Zhang S., Li Q., Hu K., Li J. (2024). Production and Characterization of Sorghum Sourdough Bread Sequentially Fermented with *Monascus purpureus* and *Lactiplantibacillus plantarum*. Food Bioprocess Technol..

[80] Mahmoud E.A.M., Kishk Y.F.M., Khalifa I., Fattah A.F.A.A. (2025). Impact of *Monascus purpureus* nano-biomass pigment-rich powder on noodle quality. J. Food Meas. Charact..

[81] Li G., Wang Y., Zhang Y., He S., Guo W. (2024). Insights into the quality and structure of dried wheat noodles as affected by *Monascus* pigments. J. Cereal Sci..

[82] Gong Z., Jiao P., Huang F., Zhang S., Zhou B. (2023). Separation and antioxidant activity of the water-soluble yellow *Monascus* pigment and its application in the preparation of functional rice noodles. LWT-Food Sci. Technol..

[83] Srianta I., Kuswardani I., Ristiarini S., Kusumawati N., Godelive L. (2022). Utilization of durian seed for *Monascus* fermentation and its application as a functional ingredient in yogurt. Bioresour. Bioprocess..

[84] Romulo A., Suliantari, Palupi N. (2017). Application of Angkak (Red Yeast Rice) Extract as Natural Red Colorant in Making of Low Fat Fruity Probiotic Yoghurt. Agric. Food Sci..

[85] Chen S.H.A., Lv B.I.N., Du X., Chen F. (2012). Pigment from red fermented rice as colouring agent for stirred skimmed milk yoghurts. Int. J. Dairy Technol..

[86] Kumura H., Ohtsuyama T., Matsusaki Y.H., Taitoh M., Koyanagi H. (2018). Application of red pigment producing edible fungi for development of a novel type of functional cheese. J. Food Process. Preserv..

[87] Xia Y., Yuan R., Weng S., Wang G., Xiong Z. (2020). Proteolysis, lipolysis, texture and sensory properties of cheese ripened by *Monascus* fumeus. Food Res. Int..

[88] Baranová M., Maa P., Burdová O., Hadbavný M., Sabolová G. (2004). Effect of natural pigment of *Monascus purpureus* on the organoleptic characters of processed cheeses. Bull. Vet. Inst. Pulawy.

[89] Tian D., Huang G., Deng X., Ren L., Yu J. (2025). The aroma compounds contributing to the characteristic flavour of ripe Pu-erh tea and their molecular mechanisms of interaction with olfactory receptors. LWT-Food Sci. Technol..

[90] Deng X., Hou Y., Zhou H., Li Y., Xue Z. (2021). Hypolipidemic, anti-inflammatory, and anti-atherosclerotic effects of tea before and after microbial fermentation. Food Sci. Nutr..

[91] Chen X., Hu Y., Zeng Z., Zhang X., Huang Y. (2025). Flavor Quality and Lipid-Lowering Function of Mixed Fermented Pu-erh Tea with Various *Monascus* Species. Foods.

[92] Zeisel S.H. (1999). Regulation of “Nutraceuticals”. Science.

[93] Affuso F., Ruvolo A., Micillo F., Sacca L., Fazio S. (2010). Effects of a nutraceutical combination (berberine, Red Yeast Rice and policosanols) on lipid levels and endothelial function randomized, double-blind, placebo-controlled study. Nutr. Metab. Cardiovasc. Dis..

[94] Patel S. (2016). Functional food Red Yeast Rice (RYR) for metabolic syndrome amelioration: A review on pros and cons. World J. Microbiol. Biotechnol..

[95] Cicero A.F.G., Fogacci F., Bove M., Veronesi M., Rizzo M. (2017). Short-Term Effects of a Combined Nutraceutical on Lipid Level, Fatty Liver Biomarkers, Hemodynamic Parameters, and Estimated Cardiovascular Disease Risk: A Double-Blind, Placebo-Controlled Randomized Clinical Trial. Adv. Ther..

[96] Peng D., Fong A., Pelt A.V. (2017). Original Research: The Effects of Red Yeast Rice Supplementation on Cholesterol Levels in Adults. Am. J. Nurs..

[97] Iskandar I., Harahap Y., Wijayanti T.R., Sandra M., Prasaja B. (2020). Efficacy and tolerability of a nutraceutical combination of Red Yeast Rice, guggulipid, and chromium picolinate evaluated in a randomized, placebo-controlled, double-blind study. Complement. Ther. Med..

[98] Tong S., Li W., Rao Y., Xiao Y., Yan Y. (2024). Microbiomics and metabolomics insights into the microbial regulation on the formation of flavor components in the traditional fermentation process of Chinese Hongqu aged vinegar. Food Sci. Hum. Wellness.

[99] Wang K., Tang N., Bian X., Geng D., Chen H. (2024). Structural characteristics, chemical compositions and antioxidant activity of melanoidins during the traditional brewing of *Monascus* vinegar. LWT-Food Sci. Technol..

[100] Meng H., Song J., Fan B., Li Y., Zhang J. (2022). *Monascus* vinegar alleviates high-fat-diet-induced inflammation in rats by regulating the NF-κB and PI3K/AKT/mTOR pathways. Food Sci. Hum. Wellness.

[101] Wang K., Li Y., Bian X., Wang C., Geng D. (2025). In vitro simulated digestive properties of *Monascus* vinegar melanoidins, and cytoprotective functions on Caco-2 cells. Food Res. Int..

[102] Meng H., Song J., Li Y., Li X., Li X. (2022). *Monascus* vinegar protects against liver inflammation in high-fat-diet rat by alleviating intestinal microbiota dysbiosis and enteritis. J. Funct. Foods.

[103] Yasuda M., Tachibana S., Kuba-Miyara M. (2012). Biochemical aspects of red koji and tofuyo prepared using *Monascus* fungi. Appl. Microbiol. Biotechnol..

[104] Flajs D., Peraica M. (2009). Toxicological properties of citrinin. Arch. Ind. Hyg. Toxicol..

[105] Ajithkumar K., Savitha A.S., Renuka M., Naik M.K. (2024). Citrinin—A Potential Mycotoxin in Food and Feed with Possible Management Strategies to Combat Its Contamination. Anti-Mycotoxin Strategies for Food and Feed.

[106] Bueno D., Istamboulie G., Muñoz R., Marty J. (2015). Determination of Mycotoxins in Food: A Review of Bioanalytical to Analytical Methods. Appl. Spectrosc. Rev..

[107] Ward N.C., Watts G.F., Eckel R.H. (2019). Response by Ward et al. to Letter Regarding Article, “Statin Toxicity: Mechanistic Insights and Clinical Implications”. Circ. Res..

[108] The European Commission (2022). Commission Regulation (EU) 2022/860 of 23 May 2022 amending Annexes III and IV to Regulation (EC) No 1925/2006 of the European Parliament and of the Council as regards food supplements containing monacolins from red yeast rice. Off. J. Eur. Union.

[109] (2015). National Food Safety Standard-Food Additive Red Yeast Rice.

[110] The European Commission (2023). Commission Regulation (EU) 2023/915 of 25 April 2023 on maximum levels for certain contaminants in food and repealing Regulation (EC) No 1881/2006. Off. J. Eur. Union.

[111] (2023). Functional Red Yeast Rice (Powder).

[112] Lin C.H., Lin H.I., Chen M.L., Lai T.T., Cao L.P. (2016). Lovastatin protects neurite degeneration in LRRK2-G2019S parkinsonism through activating the Akt/Nrf pathway and inhibiting GSK3beta activity. Hum. Mol. Genet..

[113] Yuan X., Zhong M., Huang X., Hussain Z., Ren M. (2024). Industrial Production of Functional Foods for Human Health and Sustainability. Foods.

